# Symbiotic Efficiency of Spherical and Elongated Bacteroids in the *Aeschynomene-Bradyrhizobium* Symbiosis

**DOI:** 10.3389/fpls.2019.00377

**Published:** 2019-04-02

**Authors:** Florian Lamouche, Nolwenn Bonadé-Bottino, Peter Mergaert, Benoit Alunni

**Affiliations:** Institute for Integrative Biology of the Cell, UMR 9198, CNRS/Université Paris-Sud/CEA, Université Paris Saclay, Gif-sur-Yvette, France

**Keywords:** legume-rhizobium symbiosis, bacteroid differentiation, symbiotic efficiency, flow cytometry, *Aeschynomene*, *Bradyrhizobium*

## Abstract

The legume-rhizobium symbiosis is a major supplier of fixed nitrogen in the biosphere and constitutes a key step of the nitrogen biogeochemical cycle. In some legume species belonging to the Inverted Repeat Lacking Clade (IRLC) and the Dalbergioids, the differentiation of rhizobia into intracellular nitrogen-fixing bacteroids is terminal and involves pronounced cell enlargement and genome endoreduplication, in addition to a strong loss of viability. In the *Medicago truncatula-Sinorhizobium* spp. system, the extent of bacteroid differentiation correlates with the level of symbiotic efficiency. Here, we used different physiological measurements to compare the symbiotic efficiency of photosynthetic bradyrhizobia in different *Aeschynomene* spp. (Dalbergioids) hosts inducing different bacteroid morphotypes associated with increasing ploidy levels. The strongly differentiated spherical bacteroids were more efficient than the less strongly differentiated elongated ones, providing a higher mass gain to their hosts. However, symbiotic efficiency is not solely correlated with the extent of bacteroid differentiation especially in spherical bacteroid-inducing plants, suggesting the existence of other factors controlling symbiotic efficiency.

## Introduction

Nitrogen-fixing symbioses constitute a major process in the nitrogen biogeochemical cycle, accounting for the main input of fixed nitrogen in terrestrial ecosystems (Gruber and Galloway, 2008). The interaction between rhizobia and their legume hosts leads to the formation of root or stem nodules that house the bacterial symbiont (Sprent et al., 2017). After mutual recognition of the partners through a molecular dialogue, a coordinated program of organogenesis and infection results in a mature nodule where plant symbiotic cells are filled with intracellular bacteria that differentiate into nitrogen-fixing bacteroids (Kondorosi et al., 2013).

Bacteroids do not always exhibit the same morphological features as their kin in the soil. In some legume clades such as the Inverted Repeat Lacking Clade (IRLC) to which *Medicago truncatula* belongs, bacteroids undergo a strong cell enlargement, associated to an increased membrane permeability. During this process, elongated bacteroids become polyploid and their capacity to resume growth is compromised. Their differentiation is thus considered to be terminal (Mergaert et al., 2006). These bacteroids are referred to as E-type bacteroids (i.e., E for elongated). Oppositely, in most legume clades like the ones comprising *Lotus japonicus* or common bean (*Phaseolus vulgaris*), bacteroids remain similar in size and shape to the free-living rhizobia. They also retain full capacity to resume growth when they are extracted from nodules (Mergaert et al., 2006). They are thus not terminally differentiated and are called U-type bacteroids (i.e., U for unmodified).

The phenomenon of terminal bacteroid differentiation is controlled by the host plant through the massive production of a large array of nodule-specific cysteine-rich (NCR) antimicrobial peptides (Mergaert et al., 2003; Van de Velde et al., 2010). In *M. truncatula*, more than 600 NCR genes are encoded in the genome and several of them are clustered into restricted genomic locations where gene duplication and exchanges of gene pieces between paralogs result in a dynamic growth of this gene repertoire (Alunni et al., 2007; Kondorosi et al., 2013; Pecrix et al., 2018). Remarkably, despite the large number of NCR genes suggesting *a priori* an extensive redundancy, some of the NCR genes in *M. truncatula* are essential and their mutation results in bacteroid defects and a non-functional symbiosis (Horváth et al., 2015; Kim et al., 2015; Wang et al., 2017; Yang et al., 2017). Within the IRLC, the extent of bacteroid differentiation varies depending on the host plant and is correlated with the number of NCR genes in its genome, especially with the number of cationic ones (Montiel et al., 2017).

At the functional level, it has been shown that cationic NCRs display a pore-forming activity that induces membrane permeability and ultimately microbial death (Van de Velde et al., 2010; Mikuláss et al., 2016). NCR peptides also rewire the bacterial transcriptome, showing features of a membrane stress response (Tiricz et al., 2013). The bacterial peptide transporter BacA is critical to protect the bacteroids against this antimicrobial activity of the NCRs in nodules (Haag et al., 2011). Besides their membrane-damaging activity, NCRs also have intracellular targets in rhizobia such as the cell division protein FtsZ and major chaperones like GroEL, suggesting that NCRs may interact with the bacterial machineries to induce bacterial differentiation (Farkas et al., 2014; Alunni and Gourion, 2016). In line with this hypothesis, treatment of bacterial cultures with sub-lethal doses of synthetic NCR peptides results in a partial mimicry of bacteroid differentiation, with bacterial cells that elongate and display an increased DNA content (Van de Velde et al., 2010). Finally, NCR peptides seem to interfere with the cell cycle regulatory network, as along their differentiation process, bacteroids display a major drop in the expression of the CtrA master regulator of the cell cycle and of its cognate regulon. A similar feature is also observed when synchronized bacterial cultures are treated with NCR peptides (Penterman et al., 2014; Roux et al., 2014; Pini et al., 2015).

Even if terminal bacteroid differentiation is rather the exception than the rule in the legume-rhizobium symbioses, it is found in at least five different clades (Oono and Denison, 2010). In the Dalbergioid clade, which comprises peanut (*Arachis hypogaea*) and *Aeschynomene* spp. plants, bacteroids become either elongated (E-type bacteroids) or even spherical (S-type bacteroids) depending on the host species (Staphorst and Strijdom, 1972; Bonaldi et al., 2011). These E- and S-type bacteroids are terminally differentiated, similarly as the ones found in IRLC legume nodules. In addition, the recent identification of NCR-like peptides in *Aeschynomene* plants and of CAPE peptides in peanut suggests that different plant clades use similar strategies to enforce terminal bacteroid differentiation to their rhizobial partners (Czernic et al., 2015; Karmakar et al., 2019). As NCR peptides from *Medicago* and *Aeschynomene* seem to derive from distinct ancestral genes, the most probable scenario is an evolutionary convergence between IRLC and Dalbergioids involving in each case the recruitment of immune peptides into the symbiotic process (Czernic et al., 2015). Interestingly, NCR genes are only expressed in nodules in both plant clades and seem to have functions that are now distinct from immune peptides as their expression is not pathogen responsive in *Medicago* (Guefrachi et al., 2014; Czernic et al., 2015).

Thus, plants that trigger terminal bacteroid differentiation invest a large amount of energy in maintaining a massive number of NCR genes in their genome and in the massive expression of these genes which may represent around 5% of all nodule transcripts. Moreover, ancestral state reconstruction indicated that ancestral bacteroids were of the U-morphotype. Thus the terminal bacteroid differentiation process observed in different clades of legumes must have arisen several times independently, suggesting that plants may draw some benefit from the terminal differentiated state of the bacteroids (Oono and Denison, 2010). However, what benefit plants could obtain from this costly process remained unclear (Mergaert et al., 2006). Comparison of pea (*Pisum sativum*, E-type bacteroids) and common bean (U-type bacteroids) nodules infected by *Rhizobium leguminosarum* strain A34 showed that E-type bacteroids displayed a higher symbiotic efficiency over U-type bacteroids (Oono and Denison, 2010). Similarly, *Bradyrhizobium* sp. 32H1 performed better in peanut (S-type bacteroids) than in cowpea (*Vigna unguiculata*, U-type) nodules. These studies suggest thus that bacteroid differentiation provides a higher return on investment to the host plant (S > U, E > U). In line with this hypothesis, a recent study of the symbiotic efficiency of two *M. truncatula* accessions in interaction with four *Sinorhizobium* spp. strains equally suggested the existence of a positive correlation between the extent of bacteroid differentiation (i.e., the ploidy level of the bacteroids and their cell size) and the symbiotic efficiency of the interaction (Kazmierczak et al., 2017). Using the *Aeschynomene-Bradyrhizobium* system, we recently demonstrated a higher symbiotic efficiency of *Bradyrhizobium* strain ORS285 as S-type bacteroids in *Aeschynomene indica* nodules than as E-type bacteroids in *Aeschynomene afraspera* nodules (S > E) by combining biomass measurements and C/N elemental and fluxomic analyses (Lamouche et al., 2018). In this experimental setup, S-type bacteroids display a higher ploidy than the E-type ones, suggesting that S-type bacteroids are more differentiated than E-type ones. Moreover, the formation of S-type bacteroids in *A. indica* involves an intermediate step of cell elongation before cell polarity is lost, demonstrating that the S-type bacteroids are a further developmental stage (Czernic et al., 2015).

**Figure 1:**
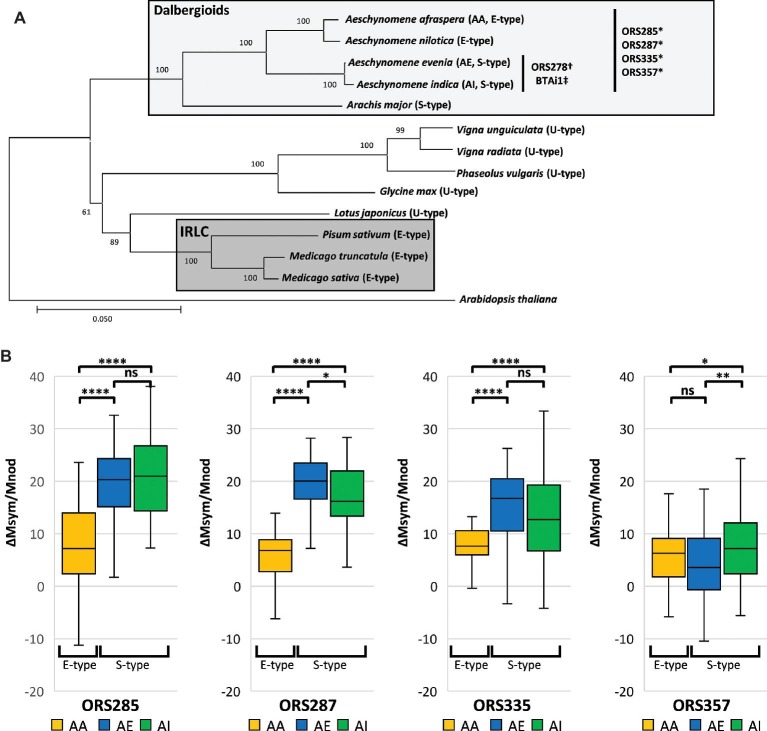
S-type bacteroids display higher symbiotic efficiency than E-type bacteroids in *Bradyrhizobium-Aeschynomene* symbioses. **(A)** Neighbor-joining tree based on nucleic *matK* sequences from Azani *et al.* (2017) of selected legume species displaying contrasting bacteroid morphotypes. The distant IRLC and Dalbergioid clade both harbor differentiated bacteroids (E- and E-/S-type, respectively) whereas other groups harbor unmodified bacteroids (U-type). Compatible associations between *Aeschynomene* spp. and *Bradyrhizobium* strains used in this study are mentioned. Strains were isolated from *: *A. afraspera*; †: *A. sensitiva*, ‡: *A. indica* (Molouba et al., 1999). **(B)** Mass gains per gram of dry nodule at 14 dpi of inoculated plants over non-inoculated (n ≥ 30) respectively elicited by *Bradyrhizobium* sp. ORS285, ORS287, ORS335, and ORS357. Significant differences after ANOVA and *post hoc* Tukey tests are indicated as ns: *p* > 0.05; *: *p* ≤ 0.05; **: *p* ≤ 0.01; ***: *p* ≤ 0.001; ****: *p* ≤ 0.0001. AA: *A. afraspera*; AE: *A. evenia*; AI: *A. indica*.

To broaden the scope of this latter comparison, we wanted to know if the higher efficiency of S-type over E-type bacteroids and if the correlation between symbiotic efficiency and the extent of bacteroid differentiation are valid in a larger sample of Dalbergioid*-Bradyrhizobium* associations (Figure 1A). We used a combination of *Bradyrhizobium* spp. that can nodulate an E-type bacteroid-forming plant (*A. afraspera*) and/or S-type bacteroid-inducing plants (*A. indica* and *Aeschynomene evenia*) (Molouba et al., 1999). We show that the more strongly differentiated S-type bacteroids are more efficient than the E-type ones. Nevertheless, at a finer scale, among the S-type bacteroids only, no correlation exists anymore between symbiotic efficiency and the level of differentiation (bacteroid ploidy level and cell size), suggesting the existence of other factors that contribute to efficiency.

## Materials and Methods

### Plant Growth and Inoculation

*Bradyrhizobium* sp. ORS278, ORS285, ORS287, ORS335, ORS357, and BTAi1 were grown at 30°C in YM medium. *A. afraspera*, *A. indica (Africa n°19)*, and *A. evenia* ssp. *evenia (n°21)* seeds were surface-sterilized by soaking the seeds for 45 min in 100% sulfuric acid for scarification. After 5 washes in sterile H_2_O, seed coat sterilization was performed for 30 min in 0.38% bleach followed by 10 washes in sterile H_2_O. Seeds were germinated in the dark on 0.7% (m/v) Kalys agar plates at 30°C (Guefrachi et al., 2015). Plantlets were transferred and grown in a semi-sterile system composed of test tubes filled with BNM medium and covered with an aluminum foil displaying a small hole for the tap root to grow through it (Bonaldi et al., 2011). Thus, the shoot is growing outside the tube whereas the root system is bathing in the culture medium. Three days after transfer, plants were inoculated with 2 ml of bacterial suspension (10^8^ cfu/ml) of the corresponding strains and cultivated during 14 days (Lamouche et al., 2018). Plants were grown at 28°C with 80% humidity under a 16 h:8 h of light:dark regimen. For 45 days post-inoculation (dpi) observations, plants were transferred in 2-L bottles and grown in the same climatic chambers.

### Microscopic Observation of Nodule Sections

Fourteen dpi nodules were harvested and 70 μm sections were produced using a Leica VT1200S vibratome before staining for 20 min with the LIVE/DEAD BacLight Bacterial viability kit (0.5 μl PI, 0.5 μl SYTO9, Thermo) in 50 mM Tris-HCl pH 7.0 buffer containing 0.01% calcofluor white M2R (Sigma). Nodule sections were rinsed and mounted in 50 mM Tris-HCl pH 7.0 buffer and imaged on a Leica SP8 confocal microscope.

### Plant Biomass Estimations

Plants (*n* ≥ 30 per condition) were collected at 14 dpi. Nodules, roots, and shoots were separated and dried at 80°C for 48 h. Dry mass was measured for the shoot (*M*_shoot_), root (*M*_root_), and nodule (*M*_nod_) plant compartments, the sum of *M*_shoot_ and *M*_root_ being called *M*_inoc_ for inoculated plants and *M*_mock_ for non-inoculated control plants. The proxy used to measure symbiotic efficiency was the total plant dry mass gain per mg of nodule, calculated as the dry mass difference between the inoculated and non-inoculated plants (Δ*M*_sym_ = *M*_inoc_ − M_mock_), divided by the nodule dry mass (i.e., Efficiency = Δ*M*_sym_/*M*_nod_). Significant differences were determined by analysis of variance followed by a *post hoc* HSD Tukey test (*p* < 0.05).

### Determination of Nitrogen Fixation Activity by Acetylene Reduction Assays

Fourteen dpi nodulated root systems were harvested and inserted into rubber-sealed glass vials containing 250 μl of sterile water. Vials were incubated for 2 h with 1 ml of acetylene at room temperature in the dark and 250 μl of gas was removed and injected in a gas chromatography system (7820A, Agilent). The area under the curve was measured for the ethylene peak, and integrated in the calculation of nitrogenase activity as previously described (Barrière et al., 2017). Dry mass measurements were performed in parallel on the same samples. More than 30 plants were used per condition.

### Flow Cytometry Analyses

For each condition, nodules were harvested on more than 15 inoculated plants and pooled to have a representative sample for flow cytometry analyses. Nodules were crushed with a mortar and a pestle in a bacteroid extraction buffer (BEB: 125 mM KCl, 50 mM Na-succinate, 50 mM TES, 0.1% BSA, pH 7.0). The nodule homogenate was centrifuged for 10 min at 100*g* to pellet plant debris. The supernatant containing the bacteroids was centrifuged for 10 min at 3600*g* to pellet the bacteroids before resupsension in a small volume of BEB. All the procedures were performed at 4°C. Bacteroids were heat-killed (for 10 min at 70°C) and stained using 50 μg ml^−1^ propidium iodide (PI). Cell size (forward scatter of the laser ray) and DNA content (level of fluorescence of the PI) were determined on a MoFlo Astrios flow cytometer and the results were analyzed using the Summit 6.2 software (Beckman Coulter).

## Results and Discussion

### *Bradyrhizobium-Aeschynomene* Associations Lead to the Formation of Functional Nodules Containing E-Type and S-Type Bacteroids Depending on the Host Plant

**Figure 2:**
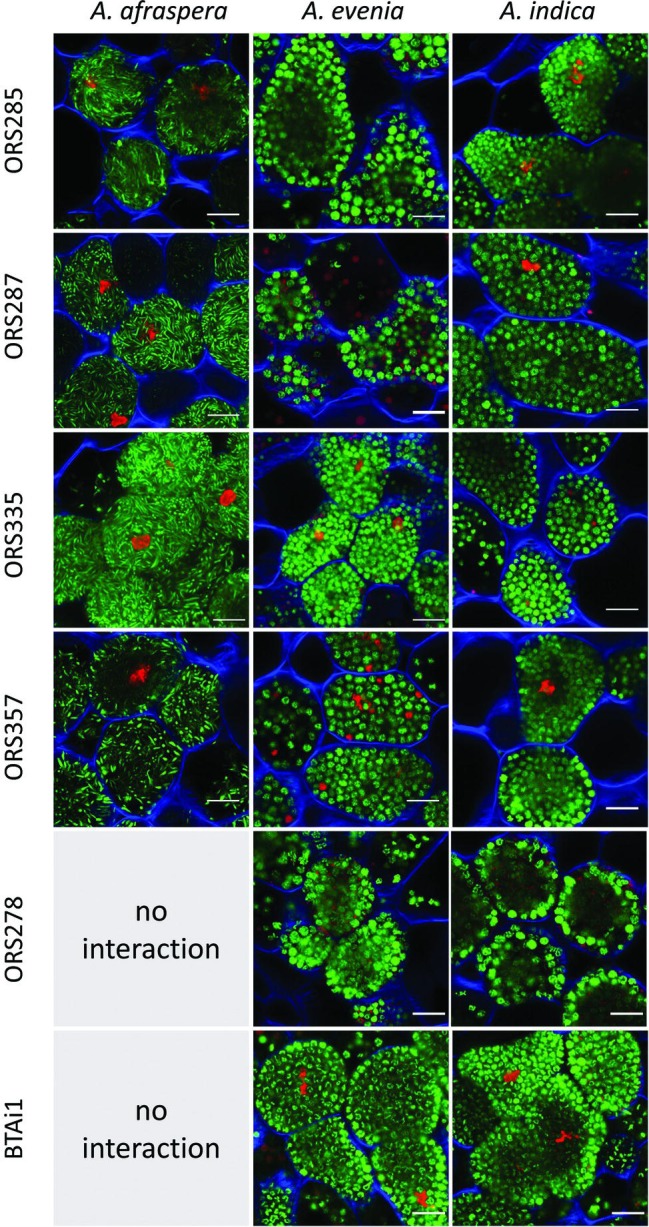
*A. indica* and *A. evenia* induce S-type bacteroids whereas *A. afraspera* induces E-type bacteroids in symbiosis with all the tested strains. Fourteen dpi nodule sections stained with the LIVE/DEAD kit (live bacteria appear green, dead bacteria and plant nuclei appear red) and calcofluor white (plant cell walls appear blue) display differentiated bacteroids within plant cells. In *A. afraspera*, bacteroids adopt an elongated morphotype, whereas in *A. evenia* and *A. indica*, bacteroids adopt a spherical morphotype regardless of the bacterial strain inoculated on the plants. Scale bars: 10 μm.

Before analyzing the symbiotic efficiency of E-type and S-type bacteroids, we first determined the functionality of the symbiosis in the 16 associations formed by *A. afraspera*, *A. evenia*, and *A. indica* and 6 *Bradyrhizobium* strains (BTAi1, ORS278, ORS285, ORS287, ORS335, and ORS357; the two first strains are unable to form nodules on *A. afraspera* because they lack the required Nod factors for nodulation of these plants (Giraud et al., 2007; Chaintreuil et al., 2013). In all cases, functional nodules were formed which displayed a pink coloration of the infected zone due to leghemoglobin production as observed in the hand-sectioned nodules, although the coloration was paler in the case of *A. evenia* nodulated by *Bradyrhizobium* sp. ORS357 (Figure S1). In agreement with this observation, confocal imaging of nodule sections showed for each interaction the presence of a large central zone composed of plant symbiotic cells infected by rhizobia (Figure S2). Bacteroid differentiation into S- or E-type bacteroids was confirmed in all plant species/bacterial strain combinations by confocal imaging at high magnification. In agreement with the bacteroid type being determined by the host, bacteroids were spherical in *A. evenia* and *A. indica*, and elongated in *A. afraspera* regardless of the bacterial strain that was inoculated (Figure 2). Thus, all the interactions tested in this study lead to the formation of functional nodules where rhizobia undergo terminal differentiation into S- or E-type bacteroids depending on the host plant. However, this gross estimation of symbiotic functionality is only qualitative and does not discriminate between weakly and highly efficient associations.

### Spherical Bacteroids Display Higher Symbiotic Efficiency Than Elongated Bacteroids in the *Bradyrhizobium*/*Aeschynomene* Symbiosis

We estimated the symbiotic efficiency for the four “generalist” strains (ORS285, ORS287, ORS335, ORS357) that can interact with both E-type bacteroid-inducing plants (*A. afraspera*) and S-type bacteroid-inducing plants (*A. evenia* and *A. indica*). To do so, we determined the gain in plant biomass attributable to the symbiosis and per investment in the symbiosis. The dry biomass of the different plant parts (i.e., shoots, roots, and nodules) was measured for both inoculated and non-inoculated plants for the different symbiotic couples at 14 dpi. The total dry mass of non-inoculated plants (*M*_mock_) was subtracted from the mass of the inoculated plants (*M*_inoc_). The resulting value (Δ*M*_sym_) thus corresponds to the biomass gained through symbiosis because it corrects the biomass of the inoculated plants by the portion of the biomass derived from the usage of nutrients stored in seeds that are different in size between *A. afraspera* (producing large seeds) and *A. indica* and *A. evenia* (producing smaller seeds). Moreover, since the onset of nitrogen fixation occurs at the same time (at 5 dpi) in the different *Aeschynomene* species, independently of the seed size and plantlet sizes (Bonaldi et al., 2011), all the tested plants fixed nitrogen during a same period (9 days) at the time of harvesting (14 dpi). Finally, the symbiotic efficiency was obtained by dividing Δ*M*_sym_ with the nodule dry mass (*M*_nod_) which is an estimation of the investment of the plant in the symbiosis. We previously used this parameter (i.e., Δ*M*_sym_/*M*_nod_) to compare *A. afraspera* and *A. indica* plants in symbiosis with *Bradyrhizobium* strain ORS285 (Lamouche et al., 2018).

The use of the plant biomass parameter for estimating the efficiency of the symbiotic process has the advantage to be a time-integrated measurement and thus to be less sensitive to temporary fluctuations that might affect instant measurements of the nitrogenase activity, for example through the often used acetylene reduction assays (ARA). We studied the relation between plant biomass parameters and the activity of nodules in the ARA using the strain ORS285 on the three studied host plants. We found that the total plant dry weight was correlated to ARA measurements in the three conditions and that Δ*M*_sym_/*M*_nod_ was correlated to the ARA measurements in two of the three hosts, with *A. evenia* showing more variability in the mass measurements (Figure S3). Thus, we considered that Δ*M*_sym_/*M*_nod_ is a good proxy for assessing the efficiency of the symbiotic process.

We found that *A. indica* and *A. evenia* plants, inducing S-type bacteroids, obtained a significantly higher benefit (*p* < 0.0001) than the *A. afraspera* plants inducing E-type bacteroids during symbiosis with all tested strains except strain ORS357 (Figure 1B). However, the latter strain did not seem to be efficiently interacting with plants inducing S-type bacteroids as suspected from the pale color of the nodule inner tissues (Figure S1). Thus overall, S-type bacteroids seem to be more efficient than the E-type ones in the Dalbergioid clade, extending our previous observations that were based on the performance of strain ORS285 in *A. afraspera* and *A. indica* (Lamouche et al., 2018). The high efficiency of the S-type bacteroids is further confirmed by the biomass of plants grown until 45 dpi when growth of *A. evenia* and *A. indica* plants had exceeded growth of *A. afraspera* plants, despite the fact that the former two species form initially smaller plantlets from smaller seeds than the latter one (Figure S4).

### Comparison of the Symbiotic Efficiency of Different Bacterial Strains on a Single Host Plant

We then considered the efficiency of a single plant genotype interacting with different symbiont strains. In all 16 interactions tested, the shoot biomass of inoculated plants was significantly higher than the non-inoculated controls, confirming that the symbiosis is a major driver of plant growth in case of nitrogen starvation (Table 1). This general gain in biomass attributable to the symbiosis indicates that the interaction is functional and fuels plant growth with nitrogen. However, the root mass was similar or even higher in non-inoculated plants than in the nodulated plants, suggesting that non-inoculated plants invest more in root growth. This is a well-known response of nitrogen-starved plants that increase their root growth to explore additional areas in the soil for nitrogen sources, resulting in a trade-off between shoot growth and root growth (Wilson, 1988). Accordingly, the shoot/root (S/R) ratio, which is often used as a proxy for the nutritional status of the plants, was higher for inoculated plants compared to control plants (Table 1). For *A. afraspera*, the differences between strains was limited, with shoot and root biomass giving similar results although the ORS285 gave the highest efficiency and ORS357 the lowest S/R ratio (Table 1).

**Table 1:** Strain-specific assessment of symbiotic efficiency in *Aeschynomene* spp.

	Shoot mass (*M*_shoot_) (mg)	Root mass (*M*_root_) (mg)	Nodule mass (*M*_nod_) (mg)	Nodule number	Total mass (*M*_tot_) (mg)	Shoot/root mass ratio	Efficiency (Δ*M*_sym_/*M*_nod_)
***A. afraspera***							
ORS285	63 (±2.38)^a^	20 (±0.9)^a^	2.6 (±0.12)^c^	24 (±1.22)^a^	86 (±3.23)^ab^	3.3 (±0.13)^a^	7.82 (±1.46)^b^
ORS287	64 (±2.12)^a^	20 (±0.88)^a^	3.14 (±0.13)^a^	20.8 (±0.84)^a^	86 (±2.97)^ab^	3.35 (±0.11)^a^	2.8 (±1.03)^a^
ORS335	71 (±2.2)^a^	22 (±0.83)^a^	3.65 (±0.14)^b^	23.03 (±0.96)^a^	97 (±3.03)^a^	3.25 (±0.06)^ab^	5.53 (±0.83)^ab^
ORS357	63 (±2.74)^a^	22 (±0.81)^a^	2.9 (±0.13)^ac^	20.63 (±1.17)^a^	87 (±3.55)^ab^	2.91 (±0.06)^b^	3.33 (±1)^a^
BTAi1	—	—	—	—	—	—	—
ORS278	—	—	—	—	—	—	—
NI	46 (±2.71)^b^	28 (±1.7)^b^	—	—	74 (±4.29)^b^	1.74 (±0.08)^c^	—
***A. evenia***							
ORS285	28 (±0.89)^ab^	8 (±0.22)^a^	0.77 (±0.03)^ab^	13.95 (±0.48)^ab^	37 (±1.08)^ab^	3.68 (±0.1)^a^	18.95 (±0.97)^a^
ORS287	30 (±0.76)^ac^	8 (±0.21)^a^	0.86 (±0.03)^ab^	15.45 (±0.43)^a^	39 (±0.96)^a^	3.86 (±0.06)^a^	20.31 (±1.06)^a^
ORS335	27 (±0.8)^b^	7 (±0.23)^ab^	0.8 (±0.03)^ab^	14.18 (±0.51)^ab^	34 (±0.98)^b^	3.89 (±0.16)^a^	15.16 (±0.95)^a^
ORS357	17 (±0.44)^d^	6 (±0.18)^b^	0.67 (±0.02)^b^	16.15 (±0.44)^a^	24 (±0.61)^c^	2.75 (±0.04)^b^	3.66 (±0.9)^b^
BTAi1	26 (±0.84)^b^	12 (±0.34)^c^	0.67 (±0.05)^b^	11.45 (±0.94)^b^	39 (±1.13)^a^	2.17 (±0.06)^c^	30.28 (±2.58)^c^
ORS278	33 (±0.93)^c^	10 (±0.26)^e^	1.39 (±0.04)^c^	34.5 (±1.11)^c^	44 (±1.18)^d^	3.33 (±0.06)^e^	16.09 (±0.52)^a^
NI	12 (±0.43)^e^	9 (±0.29)^d^	—	—	21 (±0.64)^c^	1.43 (±0.04)^d^	—
***A. indica***							
ORS285	47 (±1.62)^a^	14 (±0.42)^ac^	1.29 (±0.05)^a^	18.12 (±0.59)^a^	62 (±2)^a^	3.45 (±0.07)^a^	20.33 (±1.08)^a^
ORS287	48 (±1.57)^ab^	13 (±0.47)^abc^	1.62 (±0.05)^b^	21.32 (±0.79)^b^	63 (±2.01)^a^	3.71 (±0.07)^a^	16.62 (±0.84)^ab^
ORS335	39 (±1.33)^c^	11 (±0.37)^c^	1.27 (±0.04)^a^	17.78 (±0.56)^a^	52 (±1.69)^b^	3.61 (±0.08)^a^	12.77 (±1.02)^bc^
ORS357	33 (±1.16)^d^	12 (±0.52)^ac^	1.41 (±0.06)^ab^	21.33 (±0.72)^b^	46 (±1.59)^b^	2.8 (±0.06)^b^	7.32 (±0.9)^d^
BTAi1	32 (±1.15)^d^	14 (±0.51)^b^	1.27 (±0.05)^a^	14.83 (±0.59)^c^	48 (±1.53)^b^	2.28 (±0.06)^c^	9.39 (±1.02)^cd^
ORS278	53 (±1.81)^b^	17 (±0.63)^d^	1.88 (±0.06)^c^	27.87 (±0.79)^d^	72 (±2.4)^d^	3.08 (±0.07)^b^	19.64 (±1.08)^a^
NI	17 (±1.02)^e^	17 (±0.64)^d^	—	—	34 (±1.35)^c^	1.07 (±0.07)^d^	—

Shoot mass, root mass, nodule mass, shoot/root ratio, and symbiotic efficiency of the different *Bradyrhizobium-Aeschynomene* interactions measured at 14 dpi. NI stands for non-inoculated control plants. Mean values are displayed and the standard error is indicated between brackets. Letters represent significant differences after ANOVA and *post hoc* Tukey HSD tests (*p* < 0.05) performed independently on each plant.

More contrasted results were observed in S-type bacteroid-forming plants. While ORS278-infected plants displayed the highest total masses, they also have high nodule masses lowering the global symbiotic efficiency (Table 1). BTAi1 had the highest calculated efficiency on *A. evenia* but the S/R ratio remained low, suggesting that the plant’s nitrogen needs were not fulfilled. In this particular case, the apparent high efficiency of BTAi1 could be partially distorted by the high root mass. In addition, this contradicting observation could be also due in part to the low number of nodules formed by this strain which were however bigger than the nodules formed by the other strains (+45% in individual nodule mass as compared to ORS278).

Regarding the symbiotic efficiency on the three *Aeschynomene* species used in this study, ORS285 appeared to be the most versatile strain, with high efficiency on all tested plants. On the other hand, ORS357 seemed to be quite efficient in symbiosis with *A. afraspera,* which gives rise to E-type bacteroids, but not with the two plants harboring S-type bacteroids. Strains ORS287 and ORS335 showed intermediate values of symbiotic efficiency with the three host plants. Additionally, considering the original plant from which the bacterial strains were isolated (i.e., BTAi1 and ORS278 from S-type *Aeschynomene* and ORS285, ORS287, ORS335, and ORS357 from E-type *Aeschynomene*), we cannot link symbiotic efficiency to a co-evolution/host adaptation process that would improve the output of the symbiosis for the plant. As in the IRLC, the extent of the NCR repertoire in the plant may affect the quality of the interaction with a given strain, and on the bacterial side, the capacity to cope with NCR peptides may differ between strains. Moreover, it was shown in *M. truncatula* that a single plant genotype responds to different compatible rhizobial strains by expressing NCR genes differentially (Nallu et al., 2014; Kazmierczak et al., 2017). Similarly, *A. afraspera* expressed NCR genes to different levels when plants were inoculated by *Bradyrhizobium diazoefficiens* USDA110 or *Bradyrhizobium* sp. ORS285 (Barrière et al., 2017), suggesting that differential expression of NCR genes may take place in the different plant species/bacterial strain combinations tested here. Alternatively, the differences observed here may be due to the membrane properties of the different bacterial strains, and/or to a differential affinity of the BclA transporters present in the bacterial strains to the cocktails of NCR peptides produced by the different *Aeschynomene* plants used in this study.

### The Bacteroid Differentiation Level Is Not the Only Determinant of Symbiotic Efficiency in *Aeschynomene-Bradyrhizobium* Associations

**Figure 3:**
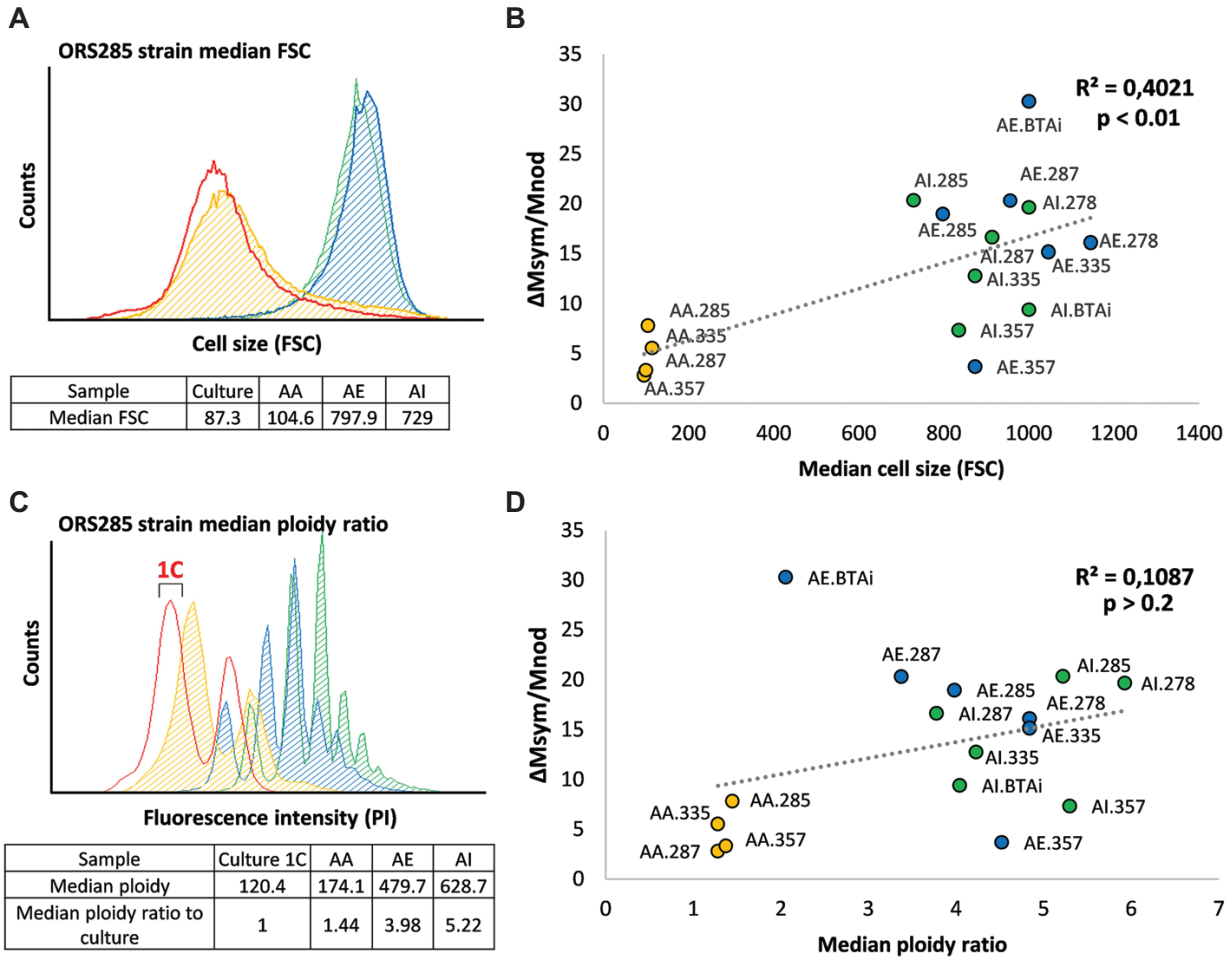
Relationship between symbiotic efficiency and bacteroid differentiation in *Bradyrhizobium-Aeschynomene* symbioses. **(A,C)** Examples of flow cytometry analysis of *Bradyrhizobium* sp. Strain ORS285 using, respectively, the forward scatter as an estimation of cell size **(A)** and propidium iodide fluorescence to estimate bacteroid ploidy **(C)** for the free-living culture reference (red), and for the *A. afraspera* (orange), *A. evenia* (blue), and *A. indica* (green) bacteroids. The fluorescence value of the 1C peak (see bracket) representing one haploid genome is the reference used to measure the median ploidy ratio of bacteroids. E-type bacteroids display a very moderate differentiation while S-type bacteroids are very different from the culture. **(B,D)** Correlation estimations between symbiotic efficiency (measured as the mass gain per gram of dry nodule) and the median cell size **(B)** or median ploidy ratios observed **(D)**. A significant correlation is only observed between bacteroid cell size and symbiotic efficiency. AA: *A. afraspera*; AE: *A. evenia*; AI: *A. indica*.

The differentiation level of the bacteroids was determined by calculating both their median ploidy ratio when compared to a bacterial culture used as reference for the haploid state (1C) (Figure 3A), as well as their cell size determined by the forward light scatter (Figure 3C). For each of the four generalist strains, we observed that both S-type bacteroids displayed higher ploidy levels, cell size, and symbiotic efficiency than E-type ones, in agreement with the previously published data on ORS285 (Figures 3B,D; Czernic et al., 2015; Lamouche et al., 2018). Taking together the results of all strains revealed a differentiation gradient from *A. afraspera*, *A. evenia* to *A. indica* bacteroids, except for ORS335 bacteroids that displayed a higher ploidy level in *A. evenia* nodules (Figure 3B). We then searched for a potential correlation between the bacteroid differentiation level and the symbiotic efficiency as observed in the IRLC (Kazmierczak et al., 2017). While increased bacteroid ploidy and cell size are correlated in our analyzed *Aeschynomene-Bradyrhizobium* associations (*R*^2^ = 0.63, *p* = 2.5e^−4^), the correlation between symbiotic efficiency and these two hallmarks of differentiation is observed only for cell size (Figure 3B; *R*^2^ = 0.40; *p* = 4.3e^−3^) and not for ploidy (Figure 3D; *R*^2^ = 0.11; *p* = 0.21). However, the latter correlation analysis is influenced by some conditions that have an unexpected and opposite relationship between efficiency and differentiation. Strain BTAi1 symbiosis with *A. evenia* was highly efficient according to our estimation while the bacteroids displayed a low endoreduplication rate, although as argued above, the efficiency estimation for strain BTAi1 in *A. evenia* might be biased. On the contrary, strain ORS357, which exhibited a low symbiotic efficiency in S-type bacteroids, underwent a high degree of differentiation.

Taken together, the correlation between bacteroid differentiation level and the symbiotic efficiency might be affected by other factors that contribute to the optimal functioning of the bacteroids. In the IRLC, it was shown that *Sinorhizobium fredii* HH103 does not undergo terminal differentiation in the NCR-producing *Glycyrrhiza uralensis* nodules although it fixes efficiently nitrogen and supports plant growth (Crespo-Rivas et al., 2016; Montiel et al., 2017). The bacterium is able to modify its LPS *in planta* possibly to cope with NCR-induced membrane stress. Similarly, some strains in our panel may somehow resist the differentiation program and investigating the structure of their LPS or other membrane components like the hopanoids (Silipo et al., 2014; Kulkarni et al., 2015) may explain partly why low differentiation levels can be associated to a high efficiency. It is also possible that the genetic differences between rhizobial strains result in the production of signals that induce strain-specific responses from the plant. As mentioned above, nodules express NCR genes to different relative levels in response to infections with different rhizobium strains. Although we did not observe any obvious signs of defense reactions in the nodules, it cannot be excluded that plants may induce an immune response to various extents against some strains, resulting in a reduced symbiotic metabolism regardless of the bacteroid differentiation level that is reached. Another aspect is that NCR peptides have been shown to have multiple intracellular targets in bacteroids in the *Medicago-Sinorhizobium* symbiosis (Farkas et al., 2014). We can speculate that similarly, *Aeschynomene* NCR peptides interact with different sets of targets in the different *Bradyrhizobium* strains affecting, thereby, the functioning of the bacteroids. Finally, as NCR peptides have a direct action on both the bacterial envelope and intracellular targets that may affect the cell metabolism, it is possible that the cocktail of NCR peptides produced by *Aeschynomene* plants inducing S-type bacteroids has an opposing effect on differentiation and symbiotic efficiency in some strains (Alunni and Gourion, 2016).

## Conclusion

In IRLC plants, it was observed that the level of bacteroid differentiation is correlated with the size of the cationic NCR repertoire (Montiel et al., 2017). In Dalbergioids, limited genomic data are available but it is not unlikely that the NCR repertoire is both quantitatively and qualitatively different between *Aeschynomene* plants inducing E- or S-type bacteroids (Czernic et al., 2015). Hence, it is possible that the NCR cocktails secreted by S-type bacteroid-forming plants exert a more stringent action on the bacteroid cell cycle and metabolism than E-type bacteroid-forming *Aeschynomene*, constraining the symbionts to a higher productivity beneficial to the host. On the other hand, while our results show that NCR-mediated differentiation affects the efficiency of the symbiotic process, additional yet unknown factors are modulating symbiotic efficiency in *Aeschynomene* spp.

## Author Contributions

FL, PM, and BA conceived the experiment(s), analyzed the result(s), and wrote the paper. FL, NB-B, and BA conducted the experiment(s). All authors reviewed the manuscript.

### Conflict of Interest Statement

The authors declare that the research was conducted in the absence of any commercial or financial relationships that could be construed as a potential conflict of interest.

## Supplementary Material

FIGURE S1 *Aeschynomene-Bradyrhizobium* associations form functional nodules. Hand-made sections of nodules show the inner part of the symbiotic organ that display a red color indicating that leghemoglobin, a marker of nodule functioning, is produced. Scale bars: 1 mm.

FIGURE S2 Functional symbiosis is associated with the presence of central nodule tissues composed of symbiotic cells infected by rhizobia. Fourteen dpi nodules were sectioned (70 µm) and stained with LIVE/DEAD kit (live/dead bacteria appear green/red respectively) and calcofluor white (plant cell walls appear blue) before confocal imaging. Scale bars: 100 µm.

FIGURE S3 Plant biomass parameters correlate with nitrogen fixation activity in symbiotic systems involving *Bradyrhizobium* sp. ORS285 and *A. afraspera* or *A. indica* and to a lesser extent with *A. evenia*. Relationship between plant total dry mass (left panel) or symbiotic efficiency (ΔM_sym_/M_nod_, right panel) and nitrogenase activity (analyzed by acetylene reduction assay and expressed as nmol C_2_H_2_ evolved per hour and per plant) was tested by calculating the correlation coefficient (R^2^) and its associated p-value for 14 dpi nodulated plants of *A. afraspera* **(A)**, *A. evenia* **(B)** and *A. indica* **(C)**.

FIGURE S4 General aspect of *Aeschynomene* plants inducing E- and S-type bacteroids at 45 dpi with ORS285 **(A)**, ORS335 **(B)**, ORS287 **(C)** or ORS357 **(D)**. The small seed producing *A. evenia* and *A. indica* developed a higher shoot length than the large seed producing *A. afraspera* regardless of the symbiotic strain. Scare bars: 5 cm.
