# Supplementary figures and images for: Symbiotic Efficiency of Spherical and Elongated Bacteroids in the Aeschynomene-Bradyrhizobium Symbiosis
Source: Front Plant Sci. 2019 Apr 2;10:377. doi: 10.3389/fpls.2019.00377 (PMC6454206; doi:10.3389/fpls.2019.00377)

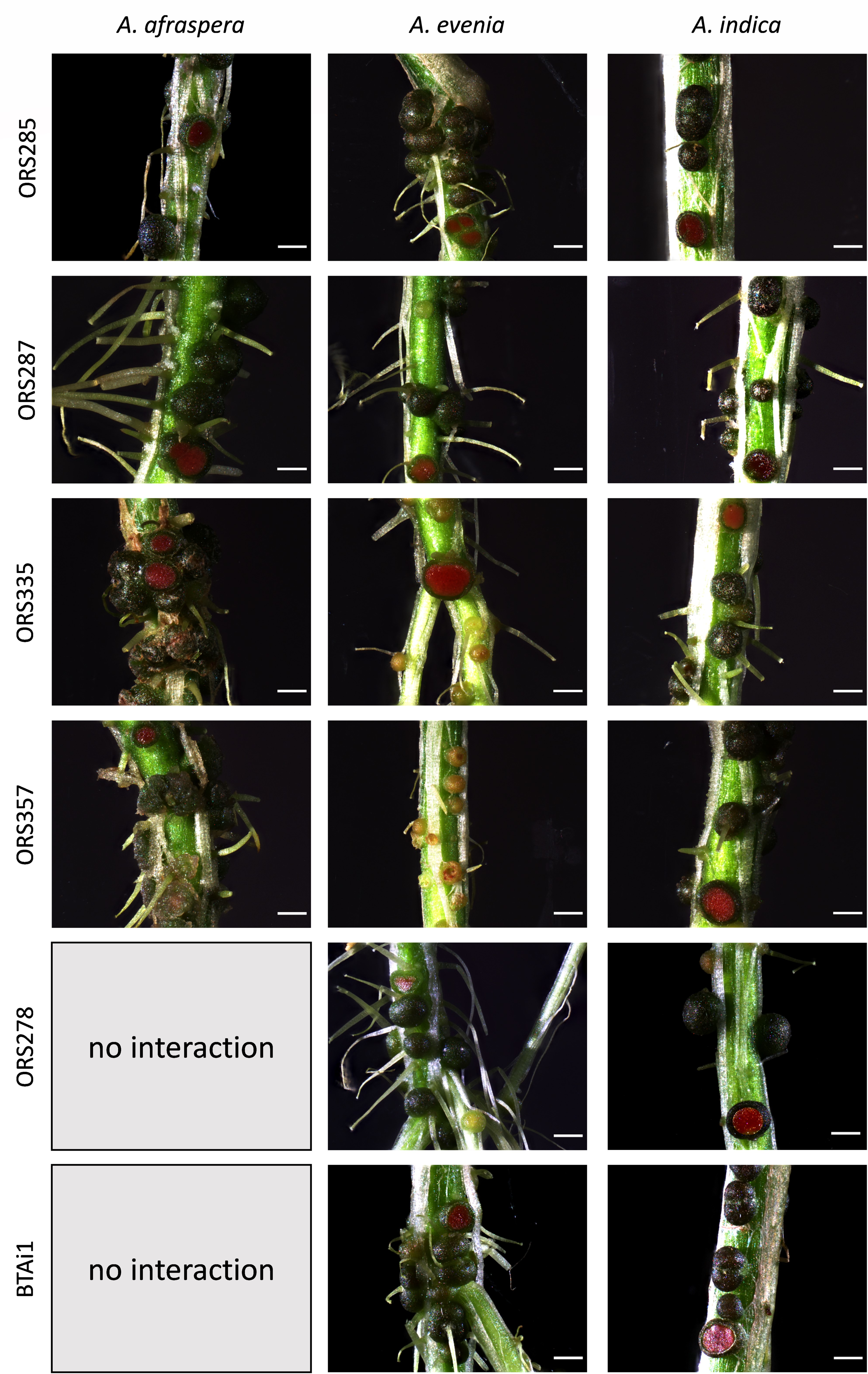

Supplement: FIGURE S1 — Aeschynomene-Bradyrhizobium associations form functional nodules. Hand-made sections of nodules show the inner part of the symbiotic organ that display a red color indicating that leghemoglobin, a marker of nodule functioning, is produced. Scale bars: 1 mm. [file Image_1.JPEG]
